# Elevated Oxidative Membrane Damage Associated with Genetic Modifiers of *Lyst*-Mutant Phenotypes

**DOI:** 10.1371/journal.pgen.1001008

**Published:** 2010-07-01

**Authors:** Colleen M. Trantow, Adam Hedberg-Buenz, Sachiyo Iwashita, Steven A. Moore, Michael G. Anderson

**Affiliations:** 1 Department of Molecular Physiology and Biophysics, The University of Iowa, Iowa City, Iowa, United States of America; 2 Department of Pathology, The University of Iowa, Iowa City, Iowa, United States of America; 3 Department of Ophthalmology and Visual Sciences, The University of Iowa, Iowa City, Iowa, United States of America; Stanford University School of Medicine, United States of America

## Abstract

LYST is a large cytosolic protein that influences the biogenesis of lysosome-related organelles, and mutation of the encoding gene, *LYST,* can cause Chediak-Higashi syndrome. Recently, *Lyst*-mutant mice were recognized to also exhibit an iris disease resembling exfoliation syndrome, a common cause of glaucoma in humans. Here, *Lyst*-mutant iris phenotypes were used in a search for genes that influence *Lyst* pathways. In a candidate gene–driven approach, albino *Lyst-*mutant mice homozygous for a mutation in *Tyr*, whose product is key to melanin synthesis within melanosomes, exhibited complete rescue of *Lyst*-mutant iris phenotypes. In a genetic background–driven approach using a DBA/2J strain of congenic mice, an interval containing *Tyrp1* enhanced *Lyst*-dependent iris phenotypes. Thus, both experimental approaches implicated the melanosome, an organelle that is a potential source of oxidative stress, as contributing to the disease phenotype. Confirming an association with oxidative damage, *Lyst* mutation resulted in genetic context–sensitive changes in iris lipid hydroperoxide levels, being lowest in albino and highest in DBA/2J mice. Surprisingly, the DBA/2J genetic background also exposed a late-onset neurodegenerative phenotype involving cerebellar Purkinje-cell degeneration. These results identify an association between oxidative damage to lipid membranes and the severity of *Lyst*-mutant phenotypes, revealing a new mechanism that contributes to pathophysiology involving LYST.

## Author Summary

LYST is a poorly understood protein involved in hereditary disease. Mutations in the encoding gene cause Chediak-Higashi syndrome, a rare lethal disease affecting multiple tissues of the body. Mutations in *Lyst* also recapitulate features of exfoliation syndrome, a common disease affecting the anterior chamber of the eye. Unfortunately, the *Lyst* gene is quite large, rendering it difficult to study by many molecular and cellular approaches. Here, we use a genetic approach in mice to identify additional genetic pathways which might modify, or prevent, the ill consequences associated with *Lyst* mutation. Our experiments demonstrate that *Lyst* mutation results in elevated levels of oxidative damage to lipid membranes. These results identify a previously unrecognized consequence of *Lyst* mutation and a modifiable pathway of potential clinical relevance in humans. Ultimately, knowledge of these events will contribute to the design of new therapeutic strategies allowing a similar alleviation of disease in humans.

## Introduction

LYST is a large cytoplasmic protein that influences several traits relevant to human health and disease [1]. Mutations in the encoding gene, *LYST*, can cause Chediak-Higashi syndrome, a rare, autosomal recessive disorder characterized by variable degrees of oculocutaneous albinism, immunodeficiency, prolonged bleeding time, and progressive neurologic dysfunction [2], [3]. *Lyst*-mutant mice also exhibit ocular defects resembling exfoliation syndrome [4], a common disease that is characterized by iris defects, fibrillar accumulations, and aberrantly dispersed pigment throughout the anterior chamber of the eye [5]. As fibrillar material and dispersed pigment accumulate in the outflow structures of the eye, intraocular pressure can become elevated and a secondary form of glaucoma often ensues. The extent to which Chediak-Higashi syndrome and exfoliation syndrome resemble each other at a mechanistic level remains to be determined, but both disease states clearly share important links to LYST.

Since the time the *Lyst* gene was initially discovered [2], [6], a cellular framework for understanding LYST function has only partially emerged. LYST is present in most tissues [7] and loss-of-function mutations lead to the enlargement of lysosome-related organelles including lysosomes, melanosomes, and platelet-dense bodies [8]. In this enlarged state, the organelles often fail to undergo normal movements [9]–[12], and exhibit altered protein components consistent with defective protein trafficking [13]–[16] as well as impaired lysosomal exocytosis leading to defects in plasma membrane repair [11]. LYST contains relatively few motifs with definitive function, thus providing limited insight into how LYST protein might contribute to these defects. Domains present in LYST include several ARM/HEAT repeats located near the amino terminus, a perilipin domain, a BEACH domain, and seven WD40 repeats located near the carboxy terminus [1]. Multiple protein-protein interactions involving LYST have been identified, including interactions with HGS, YWHAB (commonly referred to as 14-3-3), and CSNK2B [17]. Collectively, these studies suggest that LYST organizes protein-complexes important to lysosome-related organelles, perhaps through interactions with membrane domains.

Here, a genetic approach for expanding knowledge of *Lyst* function is undertaken. The goal of these experiments is to identify genetic modifiers of *Lyst*-mediated phenotypes in mice. C57BL/6J mice homozygous for the *beige-J* mutation of the *Lyst* gene (B6-*Lyst^bg-J^*) exhibit a unique iris phenotype characterized by iris stromal atrophy, pigment dispersion, dark iris color, and altered morphology of the iris pigment epithelium [4], . Because the iris is easily assayed, we reasoned that these iris phenotypes could form a convenient basis for genetic screens of *Lyst*-dependent modifiers. Two approaches are taken, one candidate-based and another based on manipulation of genetic background. Both experimental approaches implicate oxidative stress as contributing to the mechanism of disease. Testing this hypothesis directly, we found that *Lyst* mutation leads specifically to an accumulation of lipid hydroperoxides. Likely a consequence of impaired lysosomal exocytosis and a resulting failure in plasma-membrane repair, these findings implicate oxidative membrane damage as a pathological component of *Lyst*-mutant phenotypes.

## Results

### Iris Phenotypes of *Lyst*-Mutant Mice Result from Degenerative Disease

**Figure 1:**
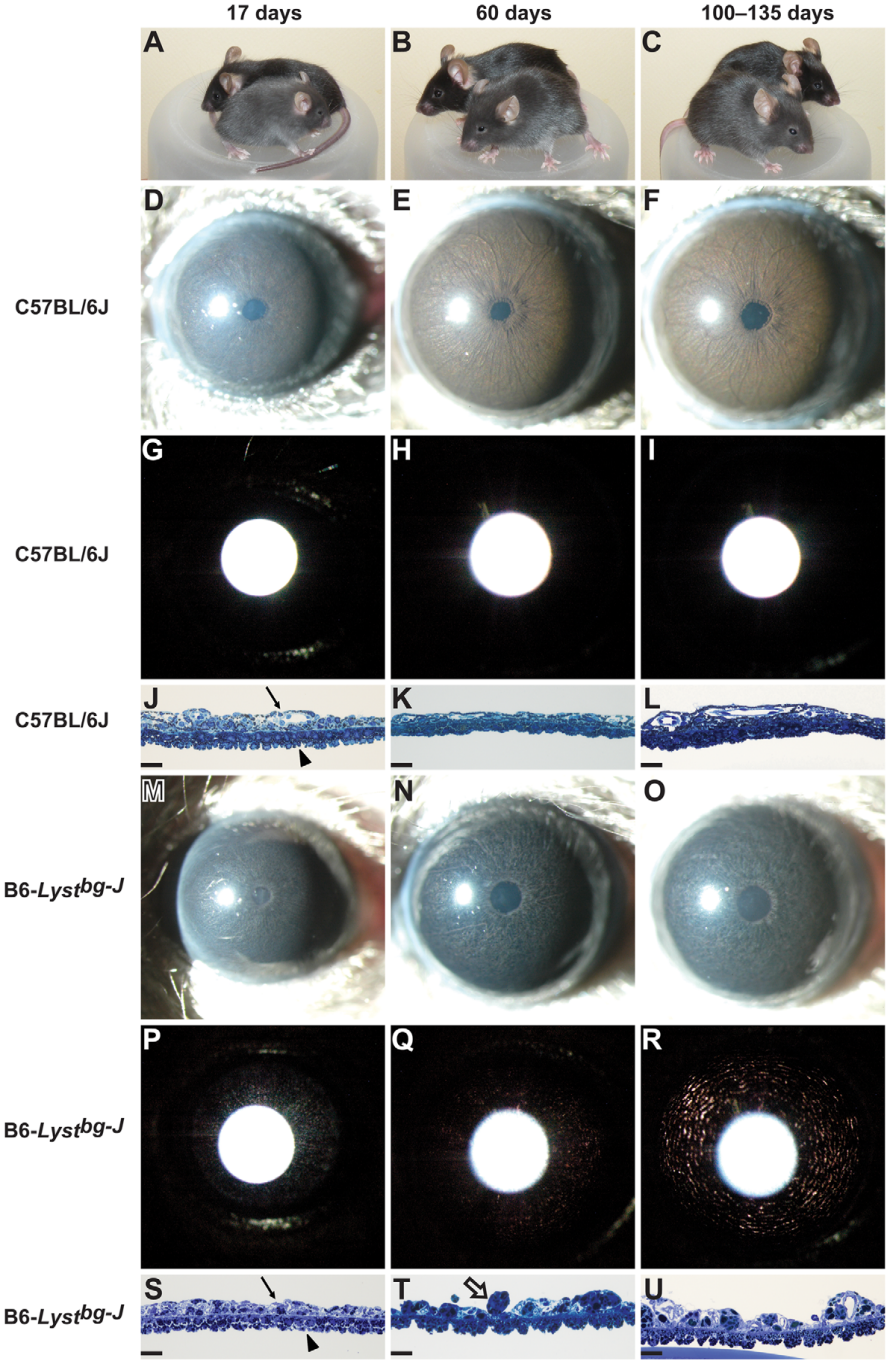
B6-*Lyst^bg-J^* mice exhibit degenerative iris phenotypes. Coat color and iris phenotypes of C57BL/6J mice and B6-*Lyst^bg-J^* mice at 17 days of age (*left column*), 60 days of age (*middle column*) and 100–135 days of age (*right column*), indicating the time course of *Lyst*-mediated phenotypes. (A–C) On the C57BL/6J genetic background, the *Lyst^bg-J^* mutation results in a dark gray coat color at all ages (B6-*Lyst^bg-J^* mice are in front, C57BL/6J mice are in back). (D–F) The C57BL/6J iris is characterized by an intact iris stroma, giving the iris a smooth appearance. The iris is accentuated by numerous underlying small vessels, which are less prominent at 17 days of age but notable by 60–135 days of age. The iris is deep sienna-brown in color. (G–I) At all ages, the C57BL/6J iris lacks transillumination defects (the bright white circle is a reflection of the photographic flash and not an iris defect). (J–L) Histologic images of the postnatal C57BL/6J iris. The iris stroma (*thin arrow*) consists of neural crest-derived melanocytes as well as dispersed collagen and extracellular matrix. The iris pigment epithelium (*arrowhead*) consists of two pigmented neural epithelium-derived cell layers. (M–O) The B6-*Lyst^bg-J^* iris is characterized by pronounced stromal atrophy and pigment dispersion, which gives the iris a granular appearing surface. Stromal atrophy is particularly pronounced in the peripupillary region, where a white ring of exposed tissue surrounding the pupil is evident. The B6-*Lyst^bg-J^* iris is distinctly dark in color, presumably as a consequence of altered melanosome structure and stromal atrophy that exposes denser pigment of the iris pigment epithelium. (P–R) B6-*Lyst^bg-J^* irides exhibit progressive transillumination defects, characterized by concentric rings of transillumination (*red areas*), which are absent at 17 days of age, minor at 60 days of age, and pronounced by 135 days of age. (S–U) The B6-*Lyst^bg-J^* iris develops normally up to day 17, with an intact iris stroma (*thin arrow*) and iris pigment epithelium (*arrowhead*). By day 60, the iris stroma is degenerating and pigment-engulfed macrophages are present (*open arrow*). The iris pigment epithelium adopts an unusual “sawtooth” morphology, in which cell-cell adhesion seems to be defective. By 100 days of age, *Lyst*-mutant phenotypes have become more striking. J–L and S–U scale bars = 25 µm.

Previously, adult B6-*Lyst^bg-J^* mice were shown to have an iris disease involving pigment dispersion and a distinct transillumination defect [4], [18]. To determine whether these phenotypes are the consequence of altered development or an early-onset degenerative disease, iris phenotypes of B6-*Lyst^bg-J^* and C57BL/6J control mice were compared throughout postnatal development (Figure 1). While the iris of C57BL/6J mice remained relatively constant with age (Figure 1A–1L), the iris of B6-*Lyst^bg-J^*mice followed a degenerative course (Figure 1M–1U; additional time points provided in Figure S1). At 17 days of age, when mice became just large enough to examine with an ophthalmic slit-lamp, the iris of B6-*Lyst^bg-J^* mice appeared relatively normal, by both slit-lamp examination (Figure 1M and 1P) and histologic analysis (Figure 1S). At 60 days of age, slit-lamp analyses indicated early signs of iris disease characterized by a dark and granular-appearing iris (Figure 1N). At this age B6-*Lyst^bg-J^* mice also exhibited minor concentric iris transillumination defects (Figure 1Q), which have previously been shown to correlate with altered morphology of the iris pigment epithelium [4]. Histologic analysis confirmed both that the iris stroma was atrophied and that the morphology of the iris pigment epithelium was altered (Figure 1T). By 100–135 days of age, these changes had become more striking (Figure 1O, 1R, and 1U), with the most notable change being that the iris transillumination defects were more pronounced. Collectively, these results indicate that iris disease in B6-*Lyst^bg-J^* mice is the consequence of an early-onset degenerative process. Having established this, we next set out to identify genetic modifiers of these *Lyst*-mutant phenotypes that might shed light on the underlying molecular mechanisms.

### Iris Degeneration in *Lyst*-Mutant Mice Is Suppressed by Tyrosinase Mutation

As in the case of B6-*Lyst^bg-J^* mice, DBA/2J mice also develop a degenerative iris disease involving iris stromal atrophy and iris transillumination defects [19], [20]. The iris disease of DBA/2J mice is caused by digenic interaction of two genes encoding proteins found within melanosomes, *Tyrp1* and *Gpnmb* [21], and can be rescued by mutations that decrease pigment production [21], [22]. To test whether pigment production is also important to the iris disease of B6-*Lyst^bg-J^* mice, genetic epistasis experiments were performed. Albino B6.*Tyr^c-2J^* mice were intercrossed with B6-*Lyst^bg-J^* mice to generate mice homozygous for both mutations on a uniform C57BL/6J genetic background (B6.*Tyr^c-2J^ Lyst^bg-J^*). The rationale for this experiment was that if pigment production contributes to *Lyst*-mutant phenotypes, B6.*Tyr^c-2J^ Lyst^bg-J^* mutant irides lacking pigment production should exhibit suppressed phenotypes.

**Figure 2:**
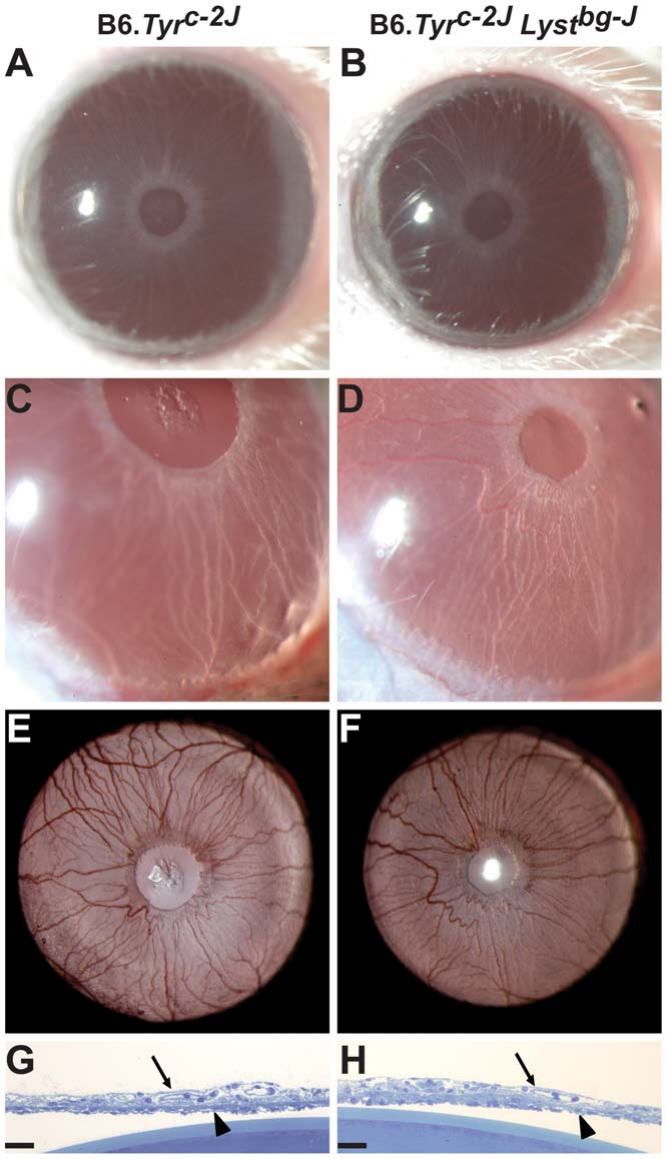
Albinism rescues *Lyst*-mutant iris phenotypes. Different views of B6.*Tyr^c-2J^* eyes (*left column*) and B6.*Tyr^c-2J^ Lyst^bg-J^* eyes (*right column*), indicating that *Lyst*-mutant iris phenotypes are completely rescued by albinism. (A, B) Broadbeam illumination reveals that mice of both genotypes have an intact iris. (C, D) At higher magnification, both genotypes have a normal appearing inferior anterior chamber that lacks the accumulations typically observed in pigmented B6-*Lyst^bg-J^* eyes. (E, F) Transilluminating illumination reveals that both genotypes have a pattern typical for albino eyes, and lack any indication of the concentric pattern characteristic of B6-*Lyst^bg-J^* eyes. (G, H) Histologic sections of B6.*Tyr^c-2J^* and B6.*Tyr^c-2J^ Lyst^bg-J^* irides are indistinguishable, both showing an intact iris stroma (*thin arrows*) and iris pigment epithelium (*arrowheads*). A portion of the lens is also visible in the bottom of both panels. Images from 3-month-old mice. Images A, C, and E are all from the same eye, as are B, D, and F. Magnifications: A, B (25×); C, D (40×, with less image reduction); E, F (25×); G, H scale bars = 25 µm.

Cohorts of B6.*Tyr^c-2J^ Lyst^bg-J^* mutant mice were generated and analyzed (Figure 2). The *Tyr^c-2J^* mutation rescued all observable *Lyst*-mediated iris phenotypes, with B6.*Tyr^c-2J^ Lyst^bg-J^* eyes indistinguishable from control B6.*Tyr^c-2J^* eyes (B6.*Tyr^c-2J^ Lyst^bg-J^*, *n* = 20 eyes at 2–5 months, 12 eyes at 9–11 months, 72 eyes at 12–19 months; B6.*Tyr^c-2J^*, *n* = 30 eyes at 2–5 months, 8 eyes at 9–11 months, 34 eyes at 12–19 months). The iris stroma of B6.*Tyr^c-2J^* and B6.*Tyr^c-2J^ Lyst^bg-J^* eyes were free of stromal atrophy (Figure 2A and 2B), with no accumulations of macrophages or debris in the anterior chamber (Figure 2C and 2D). All eyes exhibited transillumination defects typical of albino mouse eyes, with no indication of the concentric transillumination defect characteristic of *Lyst*-mutant mice (Figure 2E and 2F). Rescue was confirmed by histologic analysis of the iris (Figure 2G and 2H; additional time points in Figure S2). Together, these results identified *Tyr* as a genetic suppressor of *Lyst*-mutant iris phenotypes, and indicated that melanin production contributes to the pathological events leading to iris disease in B6-*Lyst^bg-J^* mice.

### *Lyst*-Mutant Iris Phenotypes Are Enhanced by the DBA/2J Genetic Background

**Figure 3:**
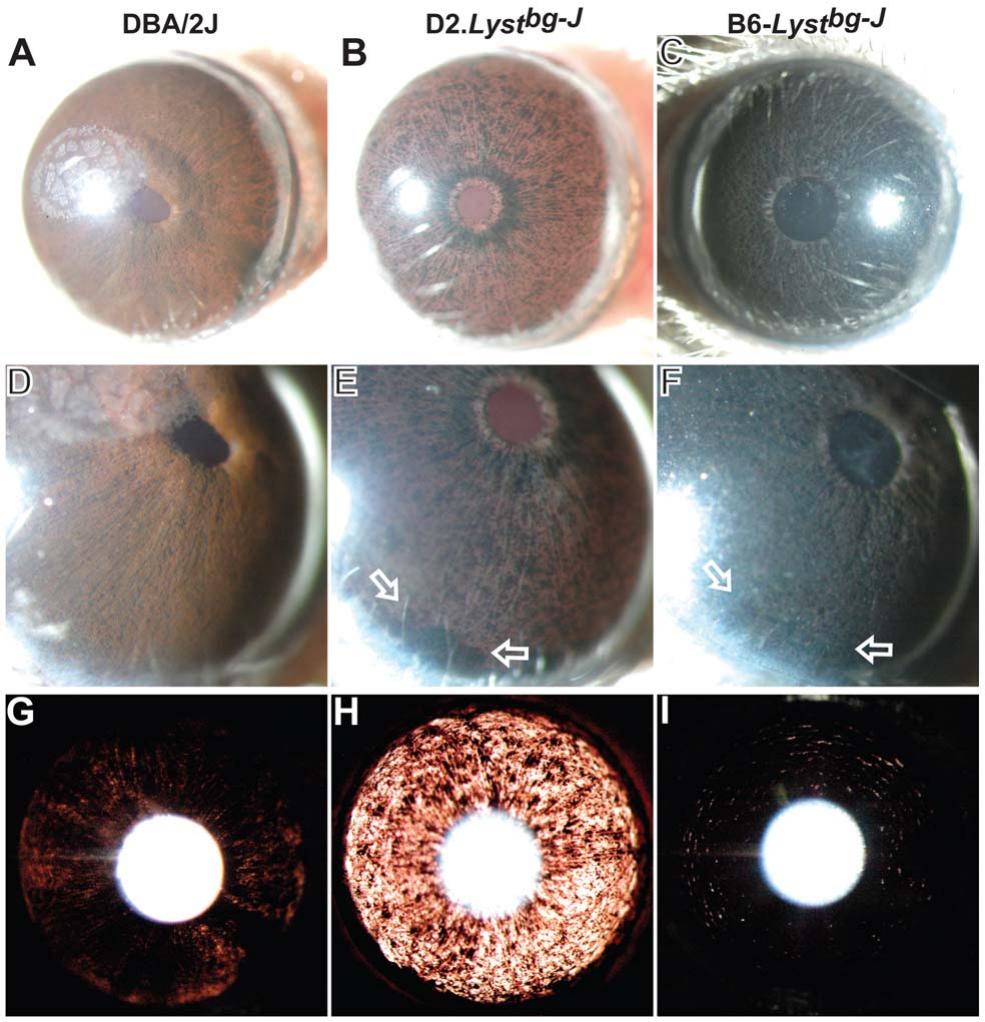
The DBA/2J genetic background enhances *Lyst*-mutant iris phenotypes. Three different slit-lamp views of DBA/2J (*left column*), D2.*Lyst^bg-J^* eyes (*middle column*), and B6-*Lyst^bg-J^* (*right column*) eyes, indicating that the DBA/2J genetic background enhances iris phenotypes caused by *Lyst* mutation. When viewed with broadbeam illumination: (A) 7-month-old DBA/2J eyes exhibit indices of mild iris disease, characterized by early signs of pigment dispersion and the swelling of peripupillary tissue, with corneal calcification typical of this strain also evident; (B) 7-month-old D2.*Lyst^bg-J^* eyes exhibit pronounced stromal atrophy and pigment dispersion, with numerous areas of depigmentation allowing light to pass freely through the iris and giving it a red hue; and (C) 9-month-old B6-*Lyst^bg-J^* eyes have pronounced stromal atrophy and pigment dispersion, but the iris remains intact overall. When viewed at higher magnification: (D) DBA/2J irides exhibit mild pigment dispersion; (E) D2.*Lyst^bg-J^* irides exhibit severe degeneration and have a granular appearance, with large accumulations of dispersed pigment and pigment engulfed macrophages visible inferiorly (*open arrows*); and (F) B6-*Lyst^bg-J^* eyes exhibit accumulations of dispersed pigment and pigment-engulfed macrophages that are visible inferiorly, but the iris is less severely degenerated. When viewed with transilluminating illumination: (G) DBA/2J eyes show mild iris transillumination defects, with subtle radial spoke-like tendencies; (H) D2.*Lyst^bg-J^* eyes have a severe transillumination defect (evident by the increased amount of red light), indicating a very atrophic iris; and (I) B6-*Lyst^bg-J^* eyes exhibit a characteristic pattern of concentric transillumination defects that are far less severe than those in eyes of D2.*Lyst^bg-J^* mice. Images in each column are all from the same eye. Magnifications: A–C (25×); D–F (40×, with less image reduction); G–I (25×).

To complement the candidate-driven search for potential *Lyst* modifiers, a genetic background-driven approach was also undertaken by creating and analyzing a congenic strain of DBA/2J mice containing the *Lyst^bg-J^* mutation (D2.*Lyst^bg-J^*). The rationale for this experiment was that *Tyrp1* mutation, *Gpnmb* mutation, or other factors from the DBA/2J genetic background might affect *Lyst*-mutant iris phenotypes. After 10 generations of backcrossing, D2.*Lyst^bg-J^* mice homozygous for the *Lyst^bg-J^* mutation were generated and assayed for relevant iris phenotypes (Figure 3). The *Lyst^bg-J^* mutation caused a lightening of the DBA/2J coat color (Figure S3). At all ages examined, the DBA/2J background enhanced *Lyst^bg-J^* ocular phenotypes (*n* = 30 eyes of D2.*Lyst^bg-J^* mice 1–7 months of age). At ages when DBA/2J mice with wild-type *Lyst* alleles exhibited only mild indices of iris abnormalities (Figure 3A, 3D, and 3G), D2.*Lyst^bg-J^* mice exhibited severe disease (Figure 3B, 3E, and 3H) that was enhanced over that in B6-*Lyst^bg-J^* mice (Figure 3C, 3F, and 3I). In D2.*Lyst^bg-J^* irides, the extent of iris stromal atrophy and iris transillumination defects was notably worsened, and resulted in large accumulations of pigment within the inferior irideocorneal angle. These results indicate that the DBA/2J genetic background enhances iris phenotypes of *Lyst^bg-J^* mice.

### *Tyrp1* Is Likely the DBA/2J-Derived Genetic Enhancer of *Lyst-*Mutant Iris Phenotypes

**Figure 4:**
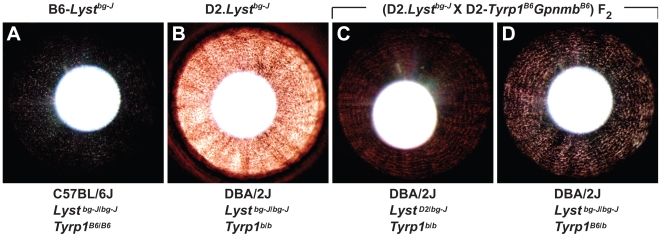
The DBA/2J-derived genetic enhancer of *Lyst-*mutant iris phenotypes maps to *Tyrp1*. Irides of mice from 4 different genetic backgrounds with the *Lyst^bg-J^* mutation indicate that *Tyrp1* mutation enhances *Lyst*-mutant iris phenotypes. (A) Subtle transillumination defect in B6-*Lyst^bg-J^* eyes. (B) Severe transillumination defect in D2.*Lyst^bg-J^* eyes. (C) Mild transillumination defect observed in (D2.*Lyst^bg-J^* X D2.*Tyrp1^B6^Gpnmb^B6^*) F_2_ mice heterozygous for the *Lyst^bg-J^* mutation but homozygous for the *Tyrp1^b^* mutation; note that the concentric pattern of defects resembles those of the *Lyst^bg-J^* phenotype. Similar results are observed in eyes of mice homozygous for wild-type *Lyst* alleles and the *Tyrp1^b^* mutation. (D) Moderate transillumination defect observed in (D2.*Lyst^bg-J^* X D2.*Tyrp1^B6^Gpnmb^B6^*) F_2_ mice heterozygous for the *Tyrp1^b^* mutation, but homozygous for the *Lyst^bg-J^* mutation; note that presence of a wild-type *Tyrp1* allele greatly alleviates the extent of transillumination in comparison to that in the D2.*Lyst^bg-J^* phenotype. Similar results are observed in eyes of mice homozygous for wild-type *Tyrp1* alleles and the *Lyst^bg-J^* mutation. Genetic background, *Lyst*, and *Tyrp1* genotype are summarized below each panel. The “B6” allele of *Tyrp1* indicates the wild-type C57BL/6J allele and the “D2” allele of *Lyst* indicates the wild-type DBA/2J allele. All mice = 1 month of age.

The identity of the DBA/2J modifier was subsequently shown to be located within a small region of mouse chromosome 4 and is likely the *Tyrp1^b^* mutation. DBA/2J mice have a known mutation in the *Tyrp1* gene [23], which similar to the *Lyst^bg-J^* mutation, also causes iris stromal atrophy [19], [21]. To directly test whether *Tyrp1* genotype influences *Lyst* phenotypes in mice, a wild-type *Tyrp1* allele was crossed onto the D2.*Lyst^bg-J^* genetic background by intercrosses with the previously described D2.*Tyrp1^B6^Gpnmb^B6^* congenic strain of mice [24]. Irides of DBA/2J mice with differing *Lyst* and *Tyrp1* genotypes were subsequently compared (Figure 4). As described above, the *Lyst^bg-J^* mutation results in a subtle, but readily detectable, pattern of iris transillumination defects on the C57BL/6J genetic background (Figure 4A), a phenotype that is greatly enhanced on the DBA/2J genetic background (Figure 4B). Among 39 (D2.*Lyst^bg-J^* X D2.*Tyrp1^B6^Gpnmb^B6^*) F_2_ progeny examined, a total of 11 mice exhibited transillumination defects of two severities. Four mice homozygous for the *Tyrp1^b^* mutation, but with at least 1 wild-type *Lyst* allele, exhibited mild transillumination defects (Figure 4C). Seven mice homozygous for the *Lyst^bg-J^* mutation, but with at least 1 wild-type *Tyrp1* allele, exhibited moderate transillumination defects (Figure 4D). The severity of transillumination defects for DBA/2J mice with wild-type *Tyrp1* were greatly reduced in comparison to those in D2.*Lyst^bg-J^* mice (compare Figure 4D to Figure 4B). *Gpnmb* genotype, which was also segregating in these crosses, had no discernable influence. Quantification based on analysis of the amount of red light present in images of these eyes (Figure S4) led to the same conclusion, transillumination defects in DBA/2J mice with wild-type *Tyrp1* were significantly reduced in comparison to those in D2.*Lyst^bg-J^* mice (*P*<0.001, Student's two-tailed *t*-test). These results map a DBA/2J-derived modifier of *Lyst* to a small (approximately 14–36 cM, ref [22]) congenic interval that encompasses the *Tyrp1* gene. Because the *Tyrp1^b^* mutation is the only known mutation within this interval in DBA/2J mice, *Tyrp1^b^* is likely to be the causative modifier. The TYRP1 protein is often affiliated with an enzymatic activity as a 5, 6-dihydroxyindole-2-carboxylic acid (DHICA) oxidase that is active in melanin synthesis [25]. However, TYRP1 has also been reported to have a catalase function, and as such could also broadly influence cellular reactions to oxidative stress [26].

### *Lyst* Mutation Results in Accumulation of Oxidatively Damaged Membranes

**Figure 5:**
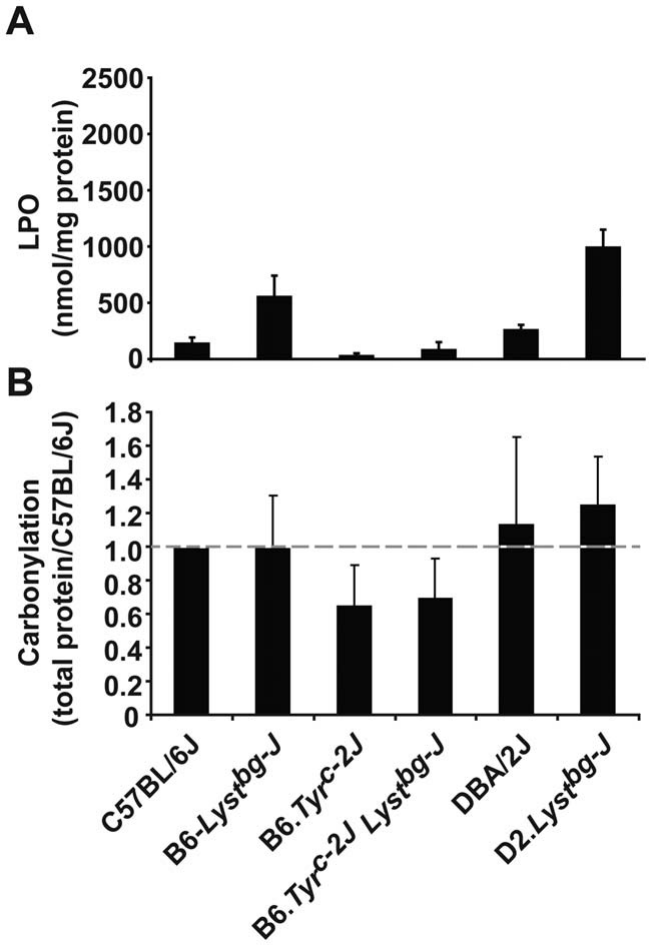
*Lyst* mutation leads to an increase in the levels of lipid hydroperoxides. (A) Lipid hydroperoxide levels in the iris according to genotype. (B) Protein carbonylation levels in the iris according to genotype. Mean ± SD; *n* = 3 for each combination of genotype and strain.

The findings of both the candidate-driven and genetic background-driven approaches suggested that *Lyst* influences oxidative stress associated with melanin synthesis. To independently test this hypothesis, lipid hydroperoxide and protein oxidation levels were measured from iris lysates of 2–3 month-old mice (Figure 5). All contexts of *Lyst* mutation resulted in significantly higher lipid hydroperoxide levels compared to strain-matched controls (Figure 5A). *Lyst* genotype, genetic background, and the interaction between *Lyst* genotype and genetic background all significantly influenced lipid hydroperoxide levels (*P*<0.001 in all comparisons, two-way ANOVA). In contrast, while genetic background significantly influenced protein carbonylation levels (*P* = 0.003, two-way ANOVA), *Lyst* genotype did not (Figure 5B). Indices of accumulated oxidative lipid damage were also observed from immunohistochemical analysis of 4-HNE localization (Figure S5). Thus, the *Lyst^bg-J^* mutation specifically altered the accumulation of oxidative damage in the membrane compartment. Importantly, the elevation in lipid hydroperoxide levels observed in the context of different genetic backgrounds mirrored the extent of disease sensitivity (B6.*Tyr^c-2J^ Lyst^bg-J^*<B6-*Lyst^bg-J^*<D2.*Lyst^bg-J^*). This correlation is consistent with the previous finding that *Lyst* mutation impairs lysosomal exocytosis, which is important for plasma membrane repair [11], and supports the notion that oxidative membrane damage contributes to the pathology of *Lyst*-mutant phenotypes.

### The DBA/2J Genetic Background Induces a *Lyst*-Mutant Neurodegenerative Phenotype

**Figure 6:**
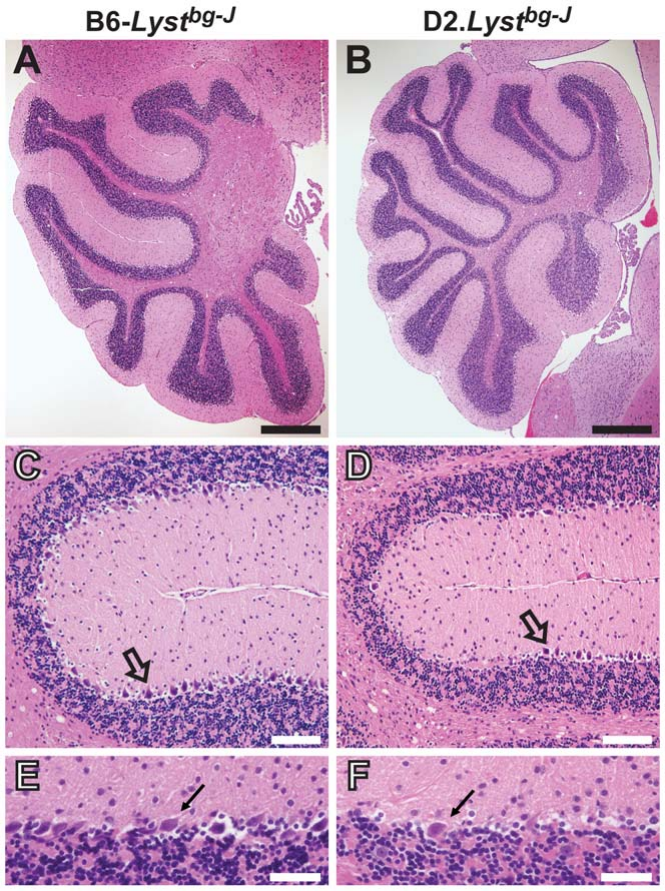
D2.*Lyst^bg-J^* mice exhibit Purkinje-cell degeneration. Mid-sagittal sections of H&E-stained cerebella revealing *Lyst*-mediated Purkinje-cell degeneration. (A, B) Low-magnification views of the cerebellar cortex, illustrating that D2.*Lyst^bg-J^* tissues exhibit no abnormalities of lobule formation or lobule size in comparison to B6-*Lyst^bg-J^* control tissues. Scale bars = 0.5 µm. (C, D) Within fissures of the cerebellar cortex, D2.*Lyst^bg-J^* tissues exhibit a loss of Purkinje cells (*open arrows*) compared to B6-*Lyst^bg-J^* control tissues. Note that Purkinje cells of B6-*Lyst^bg-J^* mice form a continuous layer of closely spaced cells, whereas there is a large gap to the left of the *open arrow* in panel D from D2.*Lyst^bg-J^* mice that is devoid of Purkinje cells. Scale bars = 100 µm. (E, F) Higher magnification images illustrating a decrease in the density of Purkinje cells (*arrows*) between the molecular (*above*) and granular-cell (*below*) layers in D2.*Lyst^bg-J^* cerebella compared to the same region in B6-*Lyst^bg-J^* age-matched controls. Scale bars = 50 µm. All mice = 17–20 months of age.

Oxidative membrane damage resulting from aberrant LYST function could have particularly important ramifications for the neurodegenerative component of Chediak-Higashi syndrome [1], [27]. Elevated levels of oxidized lipids have been observed in several neurodegenerative diseases [28]. It is possible that the same process responsible for rapid degeneration of the iris might, over a longer time frame, contribute to damage in cells that are challenged by other forms of oxidative stress, for example in aging neurons. Supporting this, extensively aged D2.*Lyst^bg-J^* mice spontaneously developed a severe tremor indicative of a neurodegenerative phenotype, whereas B6-*Lyst^bg-J^* mice did not (Video S1; *n* = 5 mice per strain, 17–20 months in age). Further histologic analysis indicated that these D2.*Lyst^bg-J^* mice exhibited Purkinje-cell degeneration (Figure 6). Although the D2.*Lyst^bg-J^* mouse cerebellum was normal in overall size and lobule morphology (Figure 6A and 6B), it consistently contained focal areas lacking Purkinje cells (Figure 6C–6F; *n* = 5 mice per strain, 17–20 months in age). Analysis of sections from the spinal cord and sciatic nerve failed to show any degenerative pathology, suggesting limited, if any, lower motor neuron or peripheral nerve involvement (Figure S6). These findings indicate that the DBA/2J genetic background also uncovered a *Lyst*-mediated phenotype in the CNS, causing a tremor likely mediated by Purkinje-cell degeneration. Because of the requirement for extensive aging, it is not yet known whether the DBA/2J-derived modifier(s) of this neurodegenerative phenotype and the degenerative iris disease are identical. However, given that *Tyrp1* is expressed in the brain (Figure S7) and is thought to exhibit catalase activity [26], this seems likely.

In order to test whether the observed Purkinje-cell degeneration also involves oxidative damage to the cell membrane, lipid hydroperoxide levels were measured in cerebellar lysates of B6-*Lyst^bg-J^* and D2.*Lyst^bg-J^* mice (*n* = 4 mice per strain, 17–20 months in age). An average 25% elevation in lipid hydroperoxides was observed in cerebella of D2.*Lyst^bg-J^* mice compared to B6-*Lyst^bg-J^* mice, but the trend was not statistically significant (*P* = 0.20, two-way ANOVA). Although no histologic defects were apparent in the cerebral cortex or brain stem (data not shown), lipid hydroperoxides in the cortex were elevated by an average of 14% (*P*<0.001, two-way ANOVA), and levels in the brain stem by 51% (*P* = 0.04, two-way ANOVA). Despite the limited statistical power of these results, they suggest that, as in the iris, the sensitivity of *Lyst*-mutant neuronal phenotypes in the CNS may involve elevated lipid hydroperoxide levels.

## Discussion

Here we have extended knowledge of *Lyst*-mediated phenotypes through studies of *Lyst* genetic modifiers. Taking advantage of iris phenotypes as a convenient assay, two genetic contexts with important modifying influences were identified. Albinism completely rescued *Lyst*-mutant iris phenotypes, and the DBA/2J genetic background enhanced them. Both results implicate melanosomes in progression of disease associated with *Lyst* mutation. Because melanin production occurring in melanosomes is a potent source of reactive oxygen species, the iris of all three strains was tested for indices of oxidative stress by measuring levels of protein and lipid oxidation. These experiments demonstrated that in pigmented cells, *Lyst* mutation specifically results in oxidative damage to lipid membranes, which correlates with the overall phenotypic severity of iris phenotypes observed among the enhancer and suppressor strains (B6.*Tyr^c-2J^ Lyst^bg-J ^*< B6-*Lyst^bg-J ^*< D2.*Lyst^bg-J^*). Thus, these experiments with *Lyst* genetic modifiers suggest that one mechanism contributing to *Lyst*-mutant phenotypes is oxidative membrane damage.

B6-*Lyst^bg-J^* mice have previously been described to exhibit multiple features of Chediak-Higashi syndrome [1], as well as an iris disease recapitulating aspects of exfoliation syndrome [4], [18]. In mice, both disease associations are characterized by changes to pigmented tissues, including coat color and iris morphology. From a mechanistic perspective, these results are directly relevant to the pathophysiology of *Lyst*-mutant defects in melanosomes. Eumelanin production occurring in melanosomes is known to be a potent source of oxidative stress [29], [30]. The mechanisms that protect melanosomes and pigment-producing cells from this insult are not well understood. Our current findings support the hypothesis that *Lyst* influences these events by modulating the repair of oxidatively damaged membranes. Exocytosis of intracellular vesicles plays an important role in plasma membrane repair [31], and experiments with cultured cells have previously demonstrated that *Lyst* mutations cause defects in lysosomal exocytosis and plasma membrane repair [11]. The oxidative membrane damage observed in the iris may well represent an accumulation caused by deficient repair. Thus, other defenses against oxidative damage to lipids are presumably overcome, leading to elevated levels of oxidatively damaged membranes and, ultimately, cellular demise [32]-[34].

The identification of *Tyrp1* as a likely modifier of *Lyst*-mutant phenotypes challenges common notions of TYRP1 function. TYRP1 is typically ascribed to function as a melanocyte-specific protein involved in melanin synthesis with DHICA oxidase activity. However, human TYRP1 appears to lack DHICA oxidase activity [35], indicating that this activity is not evolutionarily conserved. Furthermore, *Tyrp1* is not exclusively expressed in only pigment producing cells where DHICA is found. Based on our results and data provided in online databases such as the Allen Institute for Brain Science's Mouse Brain Atlas [36], *Tyrp1* is also expressed in the brain. An alternative function for TYRP1 that is consistent with our current findings is to provide catalase activity [26]. A function for TYRP1 as a catalase that influences reactive oxygen species would be consistent with the observation that the *Tyrp1^b^* mutation is associated with elevated oxidative stress, and would provide a rational explanation for its ability to enhance *Lyst*-mediated oxidative membrane damage. However, in considering potential links between *Tyrp1* and *Lyst*, it is important to point out a caveat of our current experiments. The D2-derived modifier has formally been mapped only to a congenic interval containing *Tyrp1*. Given that the *Tyrp1^b^* and *Lyst^bg-J^* mutations independently cause similar phenotypes in the iris, it is highly likely that *Tyrp1* is the causative modifier, yet it remains possible that an as yet unknown modifier exists in close proximity to this gene. Experiments testing this directly are underway.

Our current findings have important implications with respect to Chediak-Higashi syndrome. A defining component of this syndrome is progressive neurologic dysfunction [1]. Although bone-marrow transplantation can correct the immunological aspects of Chediak-Higashi syndrome and significantly extend lifespan, this treatment does not correct the neurologic aspects of the disease [37], [38]. A deeper understanding of LYST-mediated neurodegenerative phenotypes is critical for the eventual development of improved therapies for this condition. In the current analysis, a change in genetic background has uncovered a neurodegenerative phenotype involving the loss of Purkinje cells in mice with the widely utilized *Lyst^bg-J^* mutation. Our preliminary experiments suggest that, as in the case of the iris, the neuronal phenotype may involve an accumulation of oxidatively damaged membranes. Due to the large size of the neuronal cell and its expansive plasma membrane [39], neurons are likely to be in need of continuous membrane repair, and especially sensitive to defects in this process. D2.*Lyst^bg-J^* mice represent a new resource for further dissecting these mechanisms, and for testing various anti-oxidant therapies for potential benefit in mouse models of Chediak-Higashi syndrome.

Our findings also have important implications with respect to ophthalmic disease. The ocular phenotypes of B6-*Lyst^bg-J^* mice, particularly the iris transillumination defects, resemble those seen in exfoliation syndrome [4]. Several studies implicate oxidative stress as contributory to exfoliation syndrome [5], including the observation that aqueous humor from exfoliation syndrome patients has decreased levels of catalase activity [40]. The results presented here suggest that such changes are likely to be pathological. Furthermore, *LYST*, and other genes influencing oxidative stress, are suggested as candidates worthy of consideration for contributing to hereditary forms of exfoliation syndrome which is likely to also be strongly influenced by genetic modifiers [41].

Despite the existence of many *Lyst* alleles in mice, the resource that this allelic series represents has only begun to be utilized in assigning genotype-phenotype correlations. The *bg-J* mutation utilized here results from a 3-bp deletion predicted to remove a single isoleucine from the WD40 domain of the LYST protein [4]. Previous western blot analysis of cultured fibroblasts homozygous for the *bg-J* mutation failed to detect LYST protein [42], suggesting that the mutation may represent a null allele. However, this experiment has not been performed on tissues isolated directly from the mouse, nor have genetic complementation tests with a definitive null (such as a deletion or targeted mutation) been performed, leaving uncertainty regarding classification of the *bg-J* allele. Neurodegenerative phenotypes have previously been described for only one other allele (*Lyst^Ing3618^*) [43], which like the *bg-J* mutation, also disrupts the LYST WD40 domain. To our knowledge, iris phenotypes have not yet been assessed in any *Lyst* mutant strains other than those described here. Thus, it is not yet clear whether the iris and neuronal phenotypes described here will pertain to all *Lyst* alleles or might be specific to just a sub-class of mutations, though this is an issue that is addressable and worthy of follow-up.

In addition to mutations in mice, a variety of mutations relevant to LYST have been identified in other model organisms. One example is the *Drosophila* BEACH family member, *blue cheese* (*bchs*). Like LYST, the Bchs protein is predicted to be a large (400 kDa) protein containing a BEACH domain followed by a series of WD40 repeats near the C-terminus. Unlike LYST, Bchs also contains a PI(3)P-binding FYVE domain. Mutations in *bchs* result in reduced adult life span and age-related neuronal degeneration [44]. The *bchs* gene exhibits genetic interactions with genes involved in lysosomal transport and is therefore thought to encode a scaffolding protein involved in vesicle transport [45]. In motor neurons from *bchs* mutants, anterograde transport of endolysosomal vesicles toward synaptic termini is particularly affected, leading to a hypothesis that a degradative function of endolysosomal compartments at the neuromuscular junction is important in preventing neuron degeneration [46]. With respect to our current findings with *Lyst* mutant mice, these observations demonstrate that lysosomes undoubtedly make several contributions important to neuronal survival and point to the opportunity afforded by experiments with model organisms to study these events. A direct *Lyst* ortholog exists in *Drosophila* (*CG11814*), but mutant phenotypes associated with this gene have not yet been described. In the future, it will be interesting to examine the extent to which *bchs* and *CG11814* mutant phenotypes resemble each other and what additional insights might be gained by genetic studies of these genes.

In conclusion, we have performed both candidate-driven and genetic background-driven experiments to identify *Lyst* modifiers. *A priori*, the expectation would have been that modifiers of *Lyst* would logically be related to organelle biogenesis. Instead, it seems that at the level of the whole animal, oxidative damage to membranes is a highly relevant event. In our ongoing work, we intend to further test the links between *Tyrp1* and *Lyst*-mediated ophthalmic disease, and to dissect the neurodegenerative disease uncovered in D2.*Lyst^bg-J^* mice.

## Materials and Methods

### Animal Husbandry

C57BL/6J, B6-*Lyst^bg-J^*/J (abbreviated throughout as B6-*Lyst^bg-J^*), DBA/2J, and B6(Cg)-*Tyr^c-2J^*/J (abbreviated throughout as B6.*Tyr^c-2J^*) mice were obtained from The Jackson Laboratory, Bar Harbor, Maine. D2.*Tyrp1^B6^Gpnmb^B6^* mice [24] were kindly provided by Dr. Simon John of The Jackson Laboratory and subsequently bred at The University of Iowa. Unless otherwise noted, all experiments with B6-*Lyst^bg-J^* mice utilized mice homozygous for the *bg-J* mutation. All mice utilized were housed and bred at the University of Iowa Research Animal Facility. Mice were maintained on a 4% fat NIH 31 diet provided *ad libitum* and were housed in cages containing dry bedding (Cellu-dri; Shepherd Specialty Papers, Kalamazoo, MI). The environment was kept at 21°C with a 12-h light:12-h dark cycle. All animals were treated in accordance with the Association for Research in Vision and Ophthalmology Statement for the Use of Animals in Ophthalmic and Vision Research. All experimental protocols were approved by the Animal Care and Use Committee of The University of Iowa.

### Slit-Lamp Examination

Anterior chamber phenotypes were assayed using a slit-lamp (SL-D7; Topcon, Tokyo, Japan) and photodocumented using a digital camera (D100; Nikon, Tokyo, Japan). All ocular exams utilized conscious mice. Based on previous observations of *Lyst*-mutant mice [4], several traits uniformly present in adult B6-*Lyst^bg-J^* mice were followed for potential phenotypic modification. For assessment of anterior chamber phenotypes, a beam of light was shone at an angle across the eye, and the anterior chamber was examined for iris stromal atrophy, pigment dispersion, and dark iris appearance. For assessment of iris transillumination defects, a small beam of light was shone directly through the undilated pupil of the mouse and the iris was examined for the ability of reflected light to pass through diseased or depigmented areas of the iris. All photographs of like kind were taken with identical camera settings and prepared with identical image software processing. Unless otherwise noted, all slit-lamp images were collected at 25× magnification, cropped, and reduced in size.

Severity of iris transillumination defects was quantified by measuring the R-value from RGB formatted digital images. Digital images of iris transillumination defects from left and right eyes of 4 mice per genotype were analyzed using Adobe Photoshop software (Adobe Systems Inc., San Jose, CA). From 2 images per eye, 2 circular sampling windows of equivalent size, each covering approximately 5% of the measurable area of the iris, were uniformly placed (1 superior and 1 inferior) on the temporal halves of each iris image using the Elliptical Marquee tool. RGB values for the sample areas were averaged using the Average Blur Filter and R-values measured with the Eyedropper tool. In total, each genotype of mice involved analysis of 32 sample areas whose R-values were utilized in statistical analysis.

### Histology

Samples from different tissues were processed as explained below, and imaged using a light microscope (BX52; Olympus, Tokyo, Japan) equipped with a digital camera (DP72; Olympus, Tokyo, Japan).

Eyes were fixed in 2.5% gluteraldehyde in 0.1 M Na cacodylate for 16 hours, and post fixed with 1% osmium tetroxide in 0.1 M Na cacodylate buffer at room temperature for 1 hour. A series of acetone dehydrations were performed followed by infiltration with Embed-812/DDSA/NMA/DMP-30 for 24 hours. 0.5-µm sections were cut (EM UC6 ultramicrotome; Leica, Wetzler, Germany), and stained with 1% toluidine blue.

Cerebella were cut down the midline, yielding 2 hemispheres. The left cerebellar halves were fixed overnight at 4°C in 4% paraformaldehyde in 1X PBS (pH 7.4), and embedded in paraffin (Tissue Prep Paraffin Beads T565; Fisher, Pittsburgh, PA, USA). Mid-sagittal 5-µm sections were cut (Microm HM 355; Thermo Fisher, Waltham, MA, USA) and stained with hematoxylin-eosin (H&E).

Sciatic nerves were removed from the left hindlimb and fixed at 4°C in 2.5% osmotically-balanced glutaraldehyde in 0.1 M Na cacodylate buffer (pH 7.4) for at least 24 hours. Following rinses with cacodylate buffer, nerves were post fixed with 1% osmium tetroxide in 0.1 M Na cacodylate buffer at room temperature for 1 hour. Dehydration was then carried out through a series of 40-minute incubations in 25%, 50%, 75%, 90%, and 100% graded ethanol. Nerves were infiltrated overnight at room temperature, with 33%, 66%, and 100% resin (Low Viscosity Spurr Epoxy Resin; Ted Pella, Redding, CA) in propylene oxide. Specimens were embedded in resin, and 1-µm cross sections were cut (EM UC6; Leica, Wetzler, Germany) and stained with toluidine blue.

Dissected spinal columns were fixed in Bouins fixative for >1 week. Following rinses with 70% ethanol, 3-mm cross sections were cut from the cervical, thoracic, and lumbar regions of each column. The 3 cross sections from each column were embedded in paraffin (Tissue Prep Paraffin Beads T565; Fisher, Pittsburgh, PA), and 5-µm cross sections were cut (Microm HM 355; Thermo Fisher, Waltham, MA, USA) and stained with H&E.

### Strain Genotyping

The *Lyst^bg-J^* mutation results from a 3-bp deletion predicted to remove a single isoleucine from the WD40 domain of the LYST protein [4]. *Lyst* genotype was assessed by PCR amplifying a fragment of genomic DNA that flanks the causative 3-bp *bg-J* deletion [4] and assessing product lengths. To generate B6(Cg)-*Tyr^c-2J^ Lyst^bg-J^* mice (abbreviated throughout as B6.*Tyr^c-2J^ Lyst^bg-J^*), B6.*Tyr^c-2J^* mice were bred to B6-*Lyst^bg-J^* mice, and each region was bred to homozygosity. The *Tyr^c-2J^* allele is a spontaneously arising missense mutation that also influences splicing of the tyrosinase pre-mRNA, ultimately resulting in complete absence of the tyrosinase protein [47]. *Tyr* genotype was inferred from coat color. To generate congenic mice with the *Lyst^bg-J^* mutation on a DBA/2J genetic background (D2.B6-*Lyst^bg-J^*/Andm; abbreviated throughout as D2.*Lyst^bg-J^*), B6-*Lyst^bg-J^* mice were reiteratively bred to DBA/2J mice and each successive generation genotyped to select breeders heterozygous for the *Lyst^bg-J^* mutation. This process was continued for 10 generations of backcrossing. At the 10th generation, the mice were intercrossed and the *Lyst^bg-J^* mutation was bred to homozygosity. Congenic mice were genotyped with the closely linked *D13Mit17* marker and confirmed by genotyping of the causative 3 bp *bg-J* deletion. Genotypes of (D2.*Lyst^bg-J^* X D2.*Tyrp1^B6^Gpnmb^B6^*) F_2_ progeny were assessed for *Tyrp1* using the flanking markers *D4Mit327* and *D4Mit178*, and for *Gpnmb* using *D6Mit355* and *D6Mit74*.

### Reverse-Transcription PCR

For reverse-transcription PCR (RT-PCR), brains were removed and the cerebral cortex, cerebellum, and brain stem were dissected in PBS. Samples were homogenized and RNA was extracted, treated with DNase I, purified (Aurum Total RNA Mini Kit; Bio-Rad Laboratories; Hercules, CA), and converted to cDNA (iScript cDNA Synthesis Kit; Bio-Rad Laboratories; Hercules, CA). Each PCR reaction contained: 1.5 µl 10X reaction buffer (Bioline, Taunton, MA), 1.2 µl dNTPs, 0.25 µl 5′-primer (10 µM), 0.25 µl 3′-primer (10 µM), 0.25 µl MgCl_2_, 9.55 µl dH_2_O, 0.15 µl *Taq* DNA polymerase (Immolase; Bioline, Taunton, MA), and 1.5 µl cDNA (0.66 ng/ul). Primer pairs used in PCR reactions include: *Lyst* (5′-CACTGGGAGCAAGTGTGGTG-3′, 5′-TCAATTTCTGAGGGCGTGCT-3′) and *Tyrp1* (5′-TGCGATGTCTGCACTGATGA-3′, 5′-TCCAGCTGGGTTTCTCCTGA-3′). PCR conditions were: 94°C for 10 minutes, 40× (94°C for 30 seconds, 61°C for 1 minute, 72°C for 1 minute), and 72°C for 7 minutes. PCR products were analyzed on a 1% agarose gel using EtBr detection.

### Assays for Protein and Lipid Oxidation

Lipid hydroperoxide levels were measured directly (Lipid Hydroperoxide Assay; Cayman Chemical Company, Ann Arbor, MI) following the manufacturer's protocol. To measure lipid hydroperoxide levels in the iris, both irides of individual mice were dissected and sonicated in ice-cold ddH_2_O. Total protein was measured from a small aliquot (Bio-Rad Protein Assay; Bio-Rad Laboratories, Hercules, CA). The remaining sample volume was used for the extraction of lipid hydroperoxides into chloroform, and absorbance was measured following the protocol recommended by the manufacturer. Tissue lipid hydroperoxide was expressed as nmol hydroperoxide per mg of total protein. Each mouse strain was measured in biological triplicates from mice 2–4 months of age. Two-way analysis of variance (ANOVA) was employed to evaluate differences among different *Lyst* genotypes and genetic backgrounds.

For measurement of lipid hydroperoxide levels in the brain, the right cerebellar and cortical hemispheres, as well as the right half of the brain stem, were dissected and processed as described above, with the exception that samples were analyzed in two separate sessions. Two mice of each genotype were analyzed per session. The percent difference was calculated by randomly pairing B6-*Lyst^bg-J^* and D2.*Lyst^bg-J^* samples from each session. Two-way ANOVA was employed to evaluate differences among results from different genetic backgrounds and sessions.

Protein oxidation levels were measured using an immunoblotting method (OxyBlot™ Protein Oxidation Detection Kit; Millipore, Bedford, MA) following the manufacturer's protocol. Dissected irides were homogenized in lysis buffer (50 mM Tris HCl pH 7.4, 0.15 M NaCl, 1 mM EDTA, 0.1% TritonX100, 0.1% SDS, and protease inhibitors). Two aliquots of 20 µg protein each were analyzed. One aliquot was subjected to the derivatization reaction and the other served as a negative control by substituting 1× derivatization-control solution for 1× DNPH solution. Samples were denatured, derivatized, and neutralized, followed by analysis of immunoblots using an αDNP antibody. Each sample was blotted in technical triplicates (4 µl per dot) on 2 separate membranes (Immobilon-FL PVDF; Millipore, Bedford, MA) and allowed to dry completely. One membrane was stained with coomassie blue and the other was blocked (Odyssey Blocking Buffer; LI-COR Odyssey, Lincoln, NE) and incubated with αDNP primary antibody (diluted 1∶50 in blocking buffer overnight at 4°C). Following washes and incubation with secondary antibody (IRDye 680 Conjugated Gt α Rb IgG; LI-COR Odyssey, Lincoln, NE), blots were washed and quantified (LI-COR Odyssey detection system; LI-COR Odyssey, Lincoln, NE). Each sample was normalized to total protein and the amount of protein oxidation in the wild-type C57BL/6J strain. Sample analysis was repeated in 5 independent experiments. Normalized values were averaged and compared by two-way ANOVA.

### Anti-4-Hydroxy-2-Nonenal Antibody (4-HNE) Immunohistochemistry

For immunohistochemistry with 4-HNE, eyes from 6 month old mice were embedded unfixed in Optimal Cutting Temperature embedding medium (Tissue-Tek O.C.T. Compound; Sakura Finetek U.S.A., Inc., Torrance, CA); 10-µm sections were cut and sections were transferred to glass slides (CryoJane, Instrumedics, Inc., St. Louis, MO). Cryosections were air dried for 30 minutes at room temperature, fixed for 5 minutes in ice-cold acetone, again air dried for 30 minutes at room temperature, and rehydrated in PBS for 5 minutes. Sections were blocked (15 minutes at room temperature with 1 mg/ml BSA in PBS), labeled with primary antibodies (1 hour at room temperature using monoclonal mouse Anti-4-HNE antibody diluted 1∶100; Oxis International Inc., Foster City, CA), washed (three washes, 5 minutes each in PBS), and labeled with secondary antibody (1 hour at room temperature using AlexaFluor 488 conjugated antibody diluted 1∶200; Invitrogen-Molecular Probes, Carlsbad, CA). After 3 washes in PBS, the sections were mounted (ProLong Gold, Invitrogen-Molecular Probes, Carlsbad, CA), and viewed by fluorescence microscopy. All immunohistochemical experiments used assay conditions in which controls using no primary antibody lacked specific signal.

## Supporting Information

Figure S1 Additional time points in the histologic analysis of B6-*Lyst^bg-J^* irides. Histologic comparison of C57BL/6J (*left column*) and B6-*Lyst^bg-J^* (*right column*) irides throughout postnatal development. (A, B) At posnatal day 4 (P4), C57BL/6J and B6-*Lyst^bg-J^* eyes are histologically similar. Cells of the iris stroma (*thin arrows*) are visibly distinguishable from the developing trabecular meshwork and ciliary body stroma (*asterisks*). Pigmentation of the iris pigment epithelium (*arrowheads*) is complete. (C–F) At P10 and P16, C57BL/6J and B6-*Lyst^bg-J^* eyes remain histologically similar. The iris stroma and iris pigment epithelium of both genotypes are mature and fully developed. During this time, apparent spaces containing dispersed collagen and extracellular matrix become evident in both genotypes. (G, H) At P24, C57BL/6J and B6-*Lyst^bg-J^* eyes first become distinct from one another. The B6-*Lyst^bg-J^* iris stroma begins to show areas of disruption that are evident by decreased cellularity. (I, J) At P36, differences between C57BL/6J and B6-*Lyst^bg-J^* eyes become more defined. The B6-*Lyst^bg-J^* iris pigment epithelium begins to take on a “sawtooth” morphology, and pigment engulfed macrophages on the stromal surface (examples are indicated with *open arrows*, several others are unmarked) become evident as stromal atrophy continues. (K–P) At P48 and older, differences between C57BL/6J and B6-*Lyst^bg-J^* eyes become more striking and eventually appear to stabilize. Scale bars = 25 µm.
(9.44 MB TIF)

Figure S2 Additional time points in the histologic analysis of B6.*Tyr^c-2J^ Lyst^bg-J^* irides. Histologic comparison of B6.*Tyr^c-2J^* (*left column*) and B6.*Tyr^c-2J^ Lyst^bg-J^* (*right column*) irides throughout postnatal development. (A–F) Throughout early postnatal development in day P4–P16 mice, B6.*Tyr^c-2J^* and B6.*Tyr^c-2J^ Lyst^bg-J^* eyes are histologically similar. Cells of the iris stroma (*thin arrows*) are visibly distinguishable from the developing trabecular meshwork and ciliary body stroma (*asterisks*). Cells of the iris pigment epithelium (*arrowhead*) are evident. Because of the *Tyr^c-2J^* mutation, all cells lack melanin pigment. (G–J) At P24 and P36, when pigmented *Lyst^bg-J^* eyes first begin to exhibit mutant phenotypes, the iris of albino B6.*Tyr^c-2J^* and B6.*Tyr^c-2J^ Lyst^bg-J^* eyes remain histologically similar. Also unlike pigmented B6-*Lyst^bg-J^* eyes, albino B6.*Tyr^c-2J^ Lyst^bg-J^* eyes lack macrophages across the surface of the iris stroma, a further indication of an intact healthy iris. (K–P) With increasing age, the iris of B6.*Tyr^c-2J^* and B6.*Tyr^c-2J^ Lyst^bg-J^* eyes remain histologically similar, indicating the rescuing influence of the *Tyr^c-2J^* mutation to *Lyst*-mutant phenotypes. Scale bar = 25 µm.
(8.49 MB TIF)

Figure S3 Coat-color phenotypes of D2.*Lyst^bg-J^* mice. Coat color of D2.*Lyst^bg-J^* (*left*) and DBA/2J (*right*) mice. The *Lyst^bg-J^* mutation causes a lightening of the DBA/2J coat color.
(1.94 MB TIF)

Figure S4 Quantification of iris transillumination defects confirms that the DBA/2J-derived genetic enhancer of *Lyst-*mutant iris phenotypes maps to *Tyrp1*. Severity of transillumination defects quantified for multiple genotypes of mice. Transillumination defects were quantified based upon the amount of red light (*R-value*) in RGB formatted digital images of irides. As indicated in the key, images of wild-type (*WT*) eyes with no transillumination defects give rise to low R-values and eyes with mutant (*MUT*) phenotypes allowing light to pass through the iris give rise to increasing R-values. Genetic background (*BGND*), *Lyst*, and *Tyrp1* genotype are summarized below each panel. “*D2*” and “*B6*” refer to the wild-type alleles of DBA/2J or C57BL/6J mice, respectively. The (D2.*Lyst^bg-J^* X D2.*Tyrp1^B6^Gpnmb^B6^*) F_2_ cohorts include mice that are either homozygous or heterozygous for the wild-type allele of *Lyst* (5^th^ bar from left) or *Tyrp1* (6^th^ bar from left). Note that presence of a wild-type *Tyrp1* allele greatly alleviates the extent of transillumination in comparison to the D2.*Lyst^bg-J^* phenotype (*asterisk*, *P*<0.001, Student's two-tailed *t*-test). Mean ±1 SD. *n* = 8 eyes of 1-month-old mice per group.
(0.25 MB TIF)

Figure S5 *Lyst* mutation leads to increased levels of 4-HNE labeling and lipid hydroperoxide. 4-HNE labeling of C57BL/6J (*left column*) and B6-*Lyst^bg-J^* (*right column*) eyes. Identical cryosections imaged with phase-contrast (*top row*) or epifluorescence (*bottom row*) microscopy. (A, C) C57BL/6J irides have modest 4-HNE labeling of the iris stroma (*arrows*). (B, D) B6-*Lyst^bg-J^* irides have increased levels of 4-HNE labeling of the iris stroma (*arrows*), as well as pigment engulfed macrophages (*arrowhead*). All other labeling is non-specific, as determined by a negative control in which no primary antibody was used.
(4.94 MB TIF)

Figure S6 D2.*Lyst^bg-J^* mice exhibit no spinal cord or sciatic nerve degeneration. (A, B) Cross sections of the thoracic spinal cord stained with H&E. Images of the ventral grey matter illustrate similar numbers of motor neurons (*arrows*), indicating no nerve degeneration in D2.*Lyst^bg-J^* compared to B6-*Lyst^bg-J^* age-matched controls (*n* = 5 for each strain). (C, D) Cross sections of sciatic nerves stained with toluidine blue reveal a similar density of myelinated axons with no overt degeneration in D2.*Lyst^bg-J^* compared to B6-*Lyst^bg-J^* age-matched controls (*n* = 5 for each strain). Scale bars = 10 µm. All mice = 17–20 months of age.
(6.08 MB TIF)

Figure S7 *Lyst* and *Tyrp1* are expressed in the mouse brain. RT-PCR analysis shows *Lyst* expression in the cerebral cortex, cerebellum, and brain stem (*top panel*), and *Tyrp1* expression in the cerebellum and brain stem but not the cerebral cortex (*bottom panel*).
(0.20 MB TIF)

Video S1 D2.*Lyst^bg-J^* mice develop a severe tremor indicative of a neurodegenerative phenotype. Note that the D2.*Lyst^bg-J^* mouse on the left (*lighter coat color*) has a severe tremor, whereas the B6-*Lyst^bg-J^* mouse on the right (*darker coat color*) does not.
(1.08 MB WMV)
