# Supplementary figures and images for: Elevated Oxidative Membrane Damage Associated with Genetic Modifiers of Lyst-Mutant Phenotypes
Source: PLoS Genet. 2010 Jul 1;6(7):e1001008. doi: 10.1371/journal.pgen.1001008 (PMC2895641; doi:10.1371/journal.pgen.1001008)

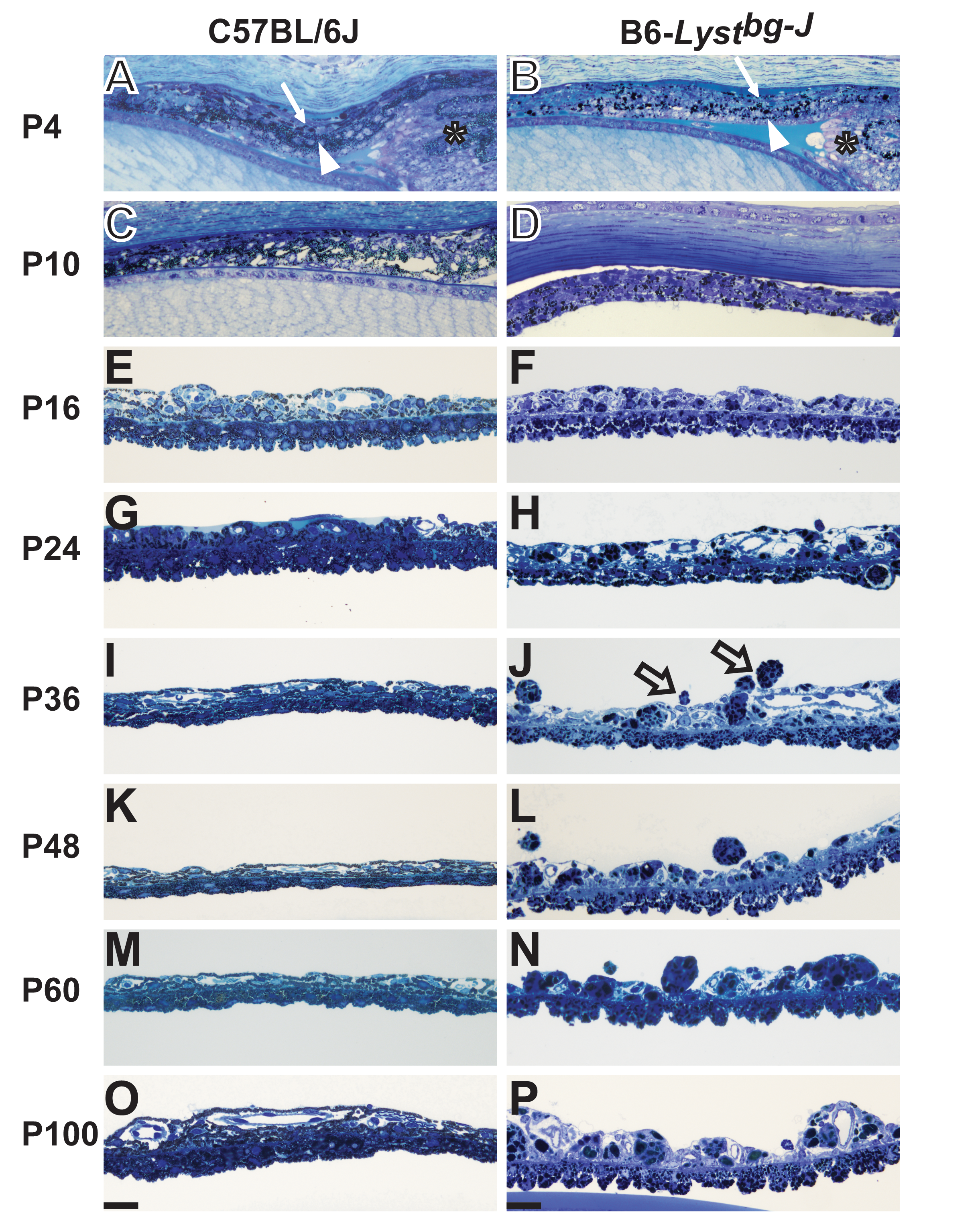

Supplement: Figure S1 — Additional time points in the histologic analysis of B6-Lystbg-J irides. Histologic comparison of C57BL/6J (left column) and B6-Lystbg-J (right column) irides throughout postnatal development. (A, B) At posnatal day 4 (P4), C57BL/6J and B6-Lystbg-J eyes are histologically similar. Cells of the iris stroma (thin arrows) are visibly distinguishable from the developing trabecular meshwork and ciliary body stroma (asterisks). Pigmentation of the iris pigment epithelium (arrowheads) is complete. (C–F) At P10 and P16, C57BL/6J and B6-Lystbg-J eyes remain histologically similar. The iris stroma and iris pigment epithelium of both genotypes are mature and fully developed. During this time, apparent spaces containing dispersed collagen and extracellular matrix become evident in both genotypes. (G, H) At P24, C57BL/6J and B6-Lystbg-J eyes first become distinct from one another. The B6-Lystbg-J iris stroma begins to show areas of disruption that are evident by decreased cellularity. (I, J) At P36, differences between C57BL/6J and B6-Lystbg-J eyes become more defined. The B6-Lystbg-J iris pigment epithelium begins to take on a “sawtooth” morphology, and pigment engulfed macrophages on the stromal surface (examples are indicated with open arrows, several others are unmarked) become evident as stromal atrophy continues. (K–P) At P48 and older, differences between C57BL/6J and B6-Lystbg-J eyes become more striking and eventually appear to stabilize. Scale bars = 25 µm. (9.44 MB TIF) [file pgen.1001008.s001.tif]

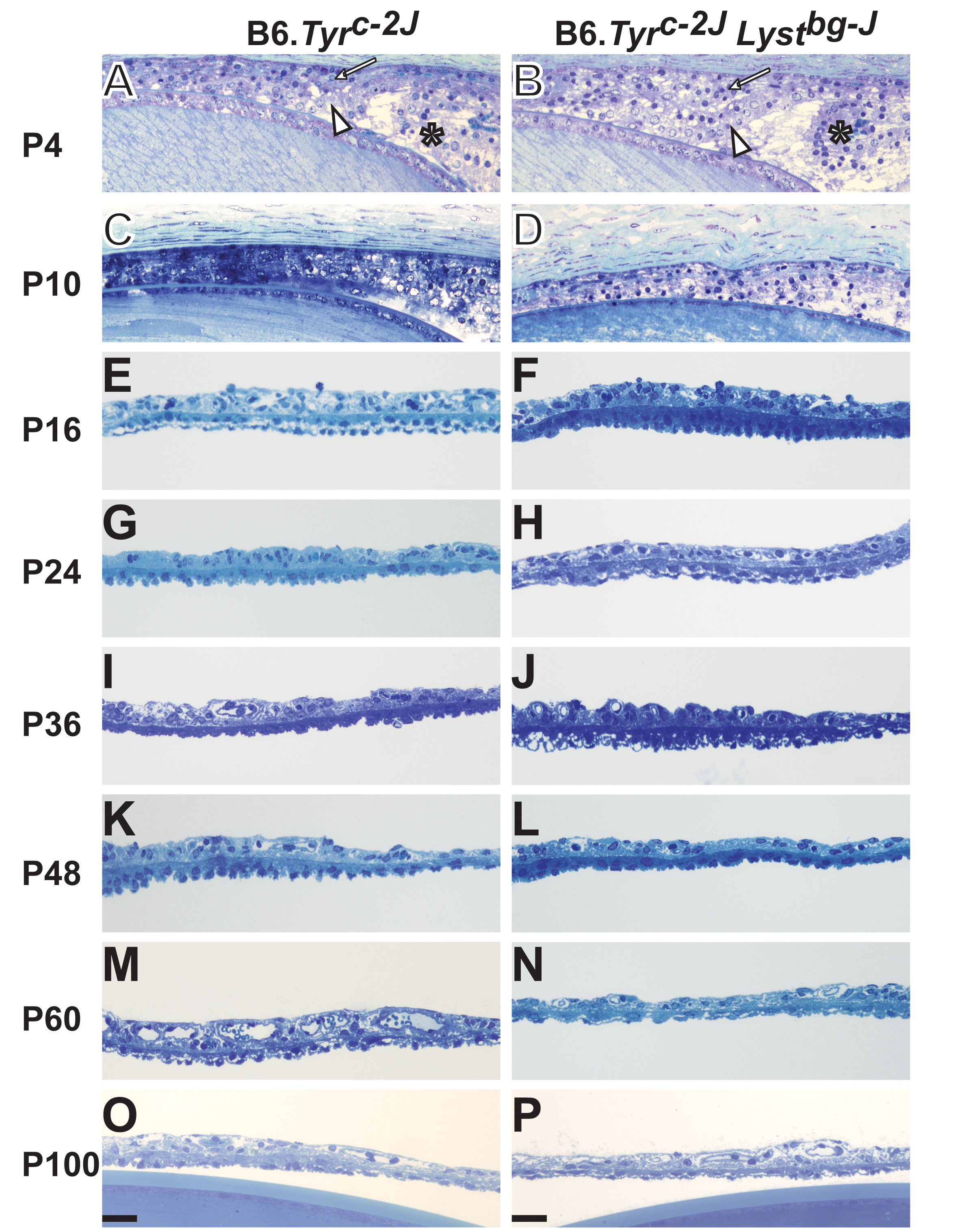

Supplement: Figure S2 — Additional time points in the histologic analysis of B6.Tyrc-2J Lystbg-J irides. Histologic comparison of B6.Tyrc-2J (left column) and B6.Tyrc-2J Lystbg-J (right column) irides throughout postnatal development. (A–F) Throughout early postnatal development in day P4–P16 mice, B6.Tyrc-2J and B6.Tyrc-2J Lystbg-J eyes are histologically similar. Cells of the iris stroma (thin arrows) are visibly distinguishable from the developing trabecular meshwork and ciliary body stroma (asterisks). Cells of the iris pigment epithelium (arrowhead) are evident. Because of the Tyrc-2J mutation, all cells lack melanin pigment. (G–J) At P24 and P36, when pigmented Lystbg-J eyes first begin to exhibit mutant phenotypes, the iris of albino B6.Tyrc-2J and B6.Tyrc-2J Lystbg-J eyes remain histologically similar. Also unlike pigmented B6-Lystbg-J eyes, albino B6.Tyrc-2J Lystbg-J eyes lack macrophages across the surface of the iris stroma, a further indication of an intact healthy iris. (K–P) With increasing age, the iris of B6.Tyrc-2J and B6.Tyrc-2J Lystbg-J eyes remain histologically similar, indicating the rescuing influence of the Tyrc-2J mutation to Lyst-mutant phenotypes. Scale bar = 25 µm. (8.49 MB TIF) [file pgen.1001008.s002.tif]

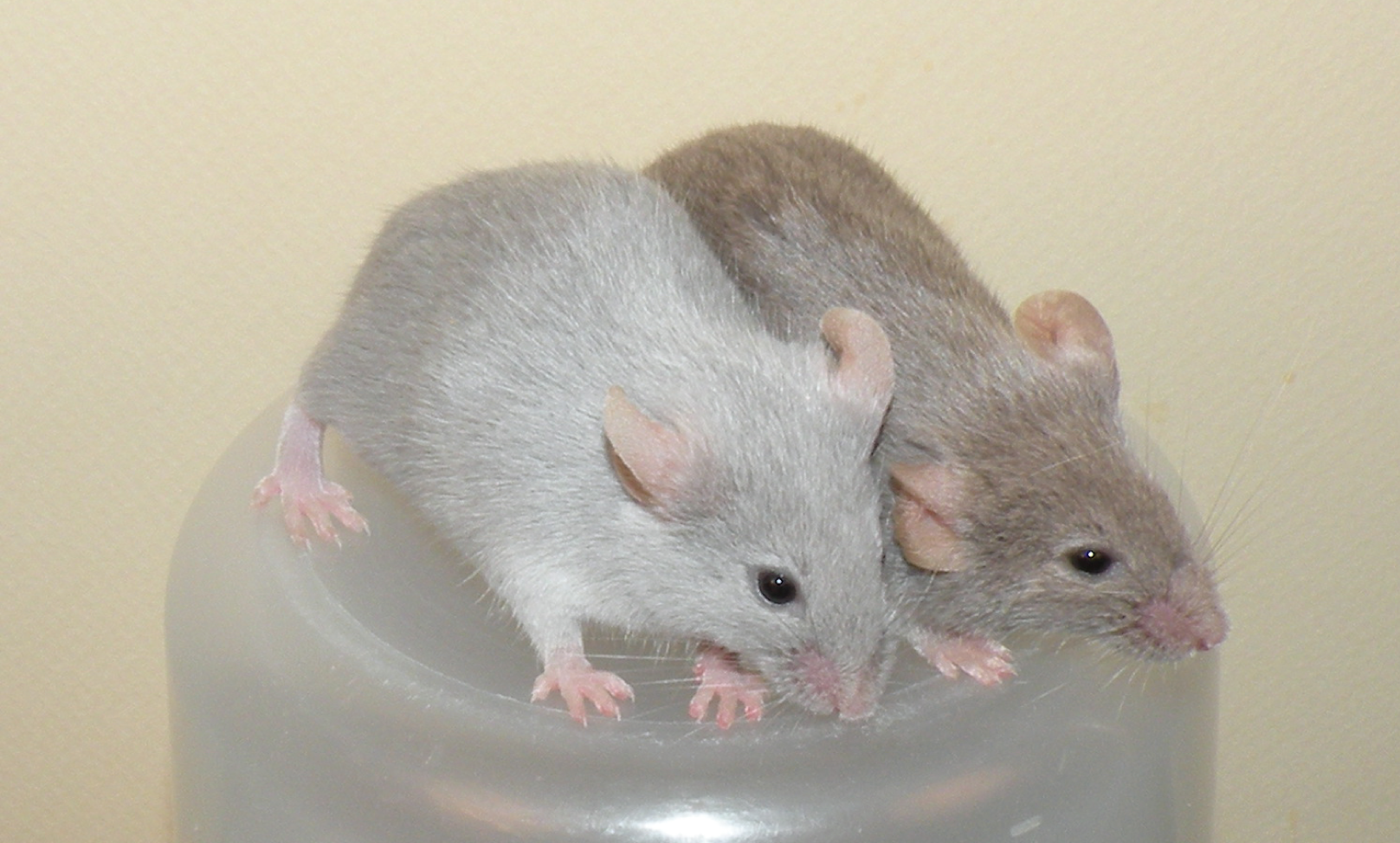

Supplement: Figure S3 — Coat-color phenotypes of D2.Lystbg-J mice. Coat color of D2.Lystbg-J (left) and DBA/2J (right) mice. The Lystbg-J mutation causes a lightening of the DBA/2J coat color. (1.94 MB TIF) [file pgen.1001008.s003.tif]

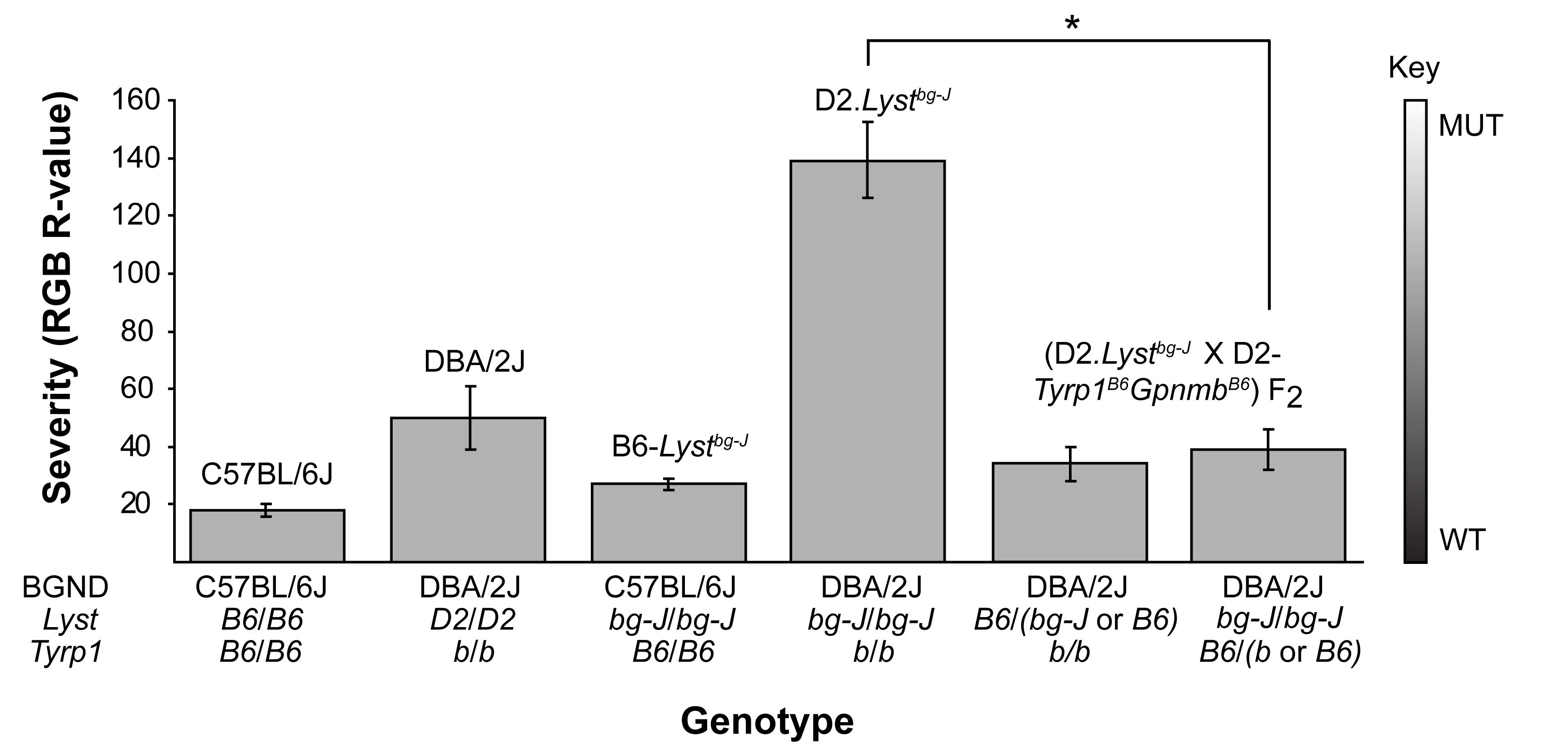

Supplement: Figure S4 — Quantification of iris transillumination defects confirms that the DBA/2J-derived genetic enhancer of Lyst-mutant iris phenotypes maps to Tyrp1. Severity of transillumination defects quantified for multiple genotypes of mice. Transillumination defects were quantified based upon the amount of red light (R-value) in RGB formatted digital images of irides. As indicated in the key, images of wild-type (WT) eyes with no transillumination defects give rise to low R-values and eyes with mutant (MUT) phenotypes allowing light to pass through the iris give rise to increasing R-values. Genetic background (BGND), Lyst, and Tyrp1 genotype are summarized below each panel. “D2” and “B6” refer to the wild-type alleles of DBA/2J or C57BL/6J mice, respectively. The (D2.Lystbg-J X D2.Tyrp1B6GpnmbB6) F2 cohorts include mice that are either homozygous or heterozygous for the wild-type allele of Lyst (5th bar from left) or Tyrp1 (6th bar from left). Note that presence of a wild-type Tyrp1 allele greatly alleviates the extent of transillumination in comparison to the D2.Lystbg-J phenotype (asterisk, P<0.001, Student's two-tailed t-test). Mean ±1 SD. n = 8 eyes of 1-month-old mice per group. (0.25 MB TIF) [file pgen.1001008.s004.tif]

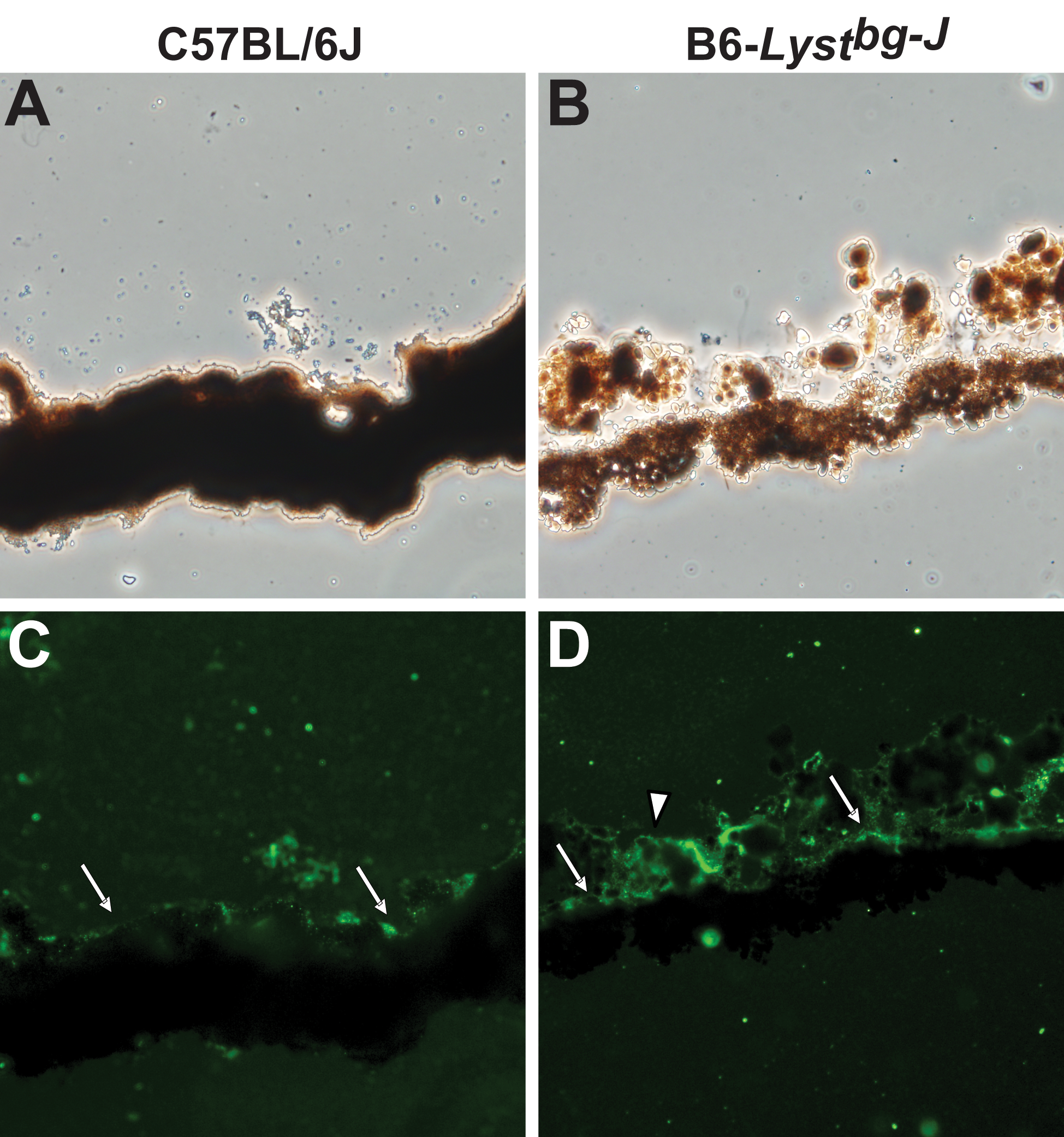

Supplement: Figure S5 — Lyst mutation leads to increased levels of 4-HNE labeling and lipid hydroperoxide. 4-HNE labeling of C57BL/6J (left column) and B6-Lystbg-J (right column) eyes. Identical cryosections imaged with phase-contrast (top row) or epifluorescence (bottom row) microscopy. (A, C) C57BL/6J irides have modest 4-HNE labeling of the iris stroma (arrows). (B, D) B6-Lystbg-J irides have increased levels of 4-HNE labeling of the iris stroma (arrows), as well as pigment engulfed macrophages (arrowhead). All other labeling is non-specific, as determined by a negative control in which no primary antibody was used. (4.94 MB TIF) [file pgen.1001008.s005.tif]

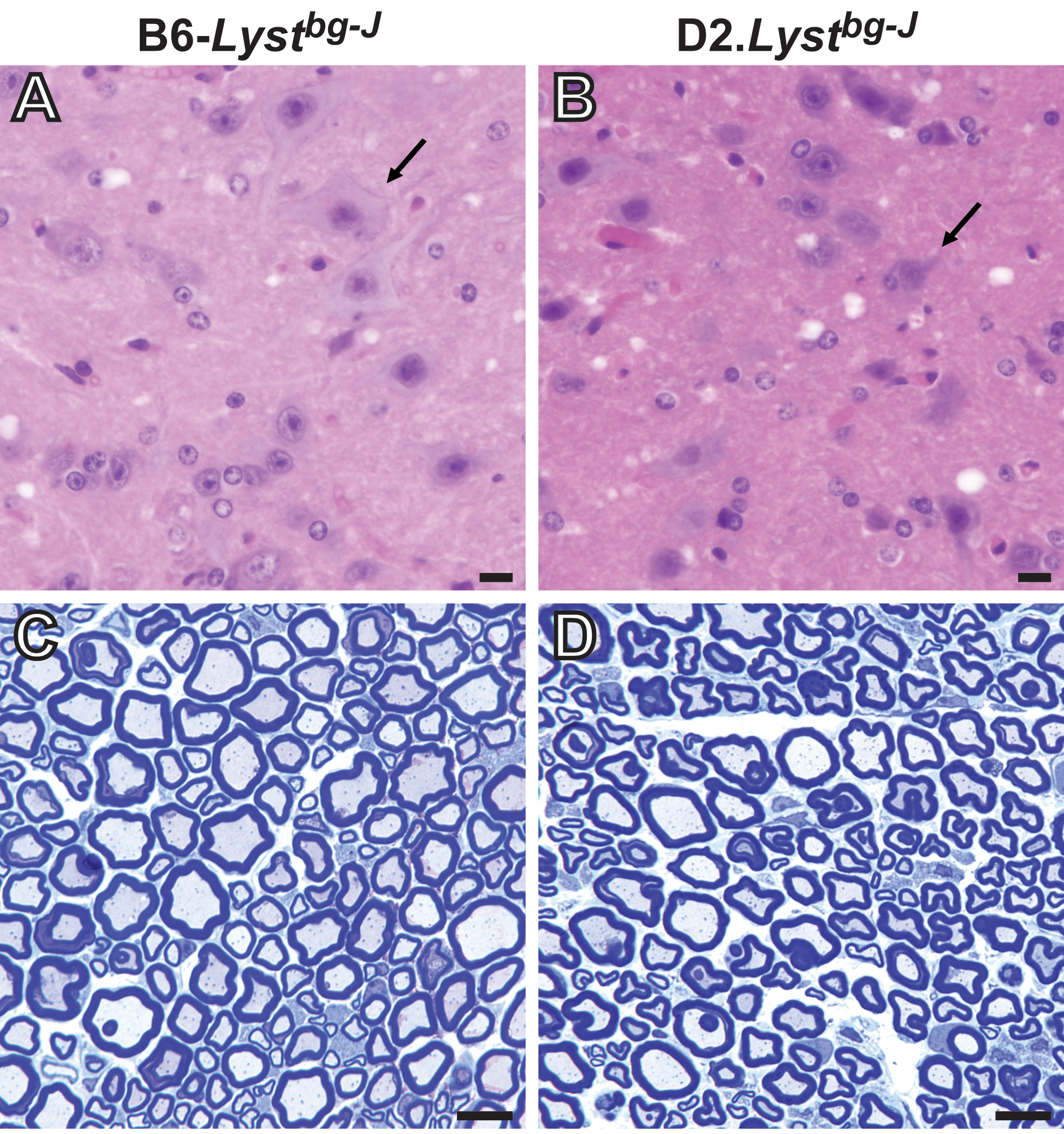

Supplement: Figure S6 — D2.Lystbg-J mice exhibit no spinal cord or sciatic nerve degeneration. (A, B) Cross sections of the thoracic spinal cord stained with H&E. Images of the ventral grey matter illustrate similar numbers of motor neurons (arrows), indicating no nerve degeneration in D2.Lystbg-J compared to B6-Lystbg-J age-matched controls (n = 5 for each strain). (C, D) Cross sections of sciatic nerves stained with toluidine blue reveal a similar density of myelinated axons with no overt degeneration in D2.Lystbg-J compared to B6-Lystbg-J age-matched controls (n = 5 for each strain). Scale bars = 10 µm. All mice = 17–20 months of age. (6.08 MB TIF) [file pgen.1001008.s006.tif]

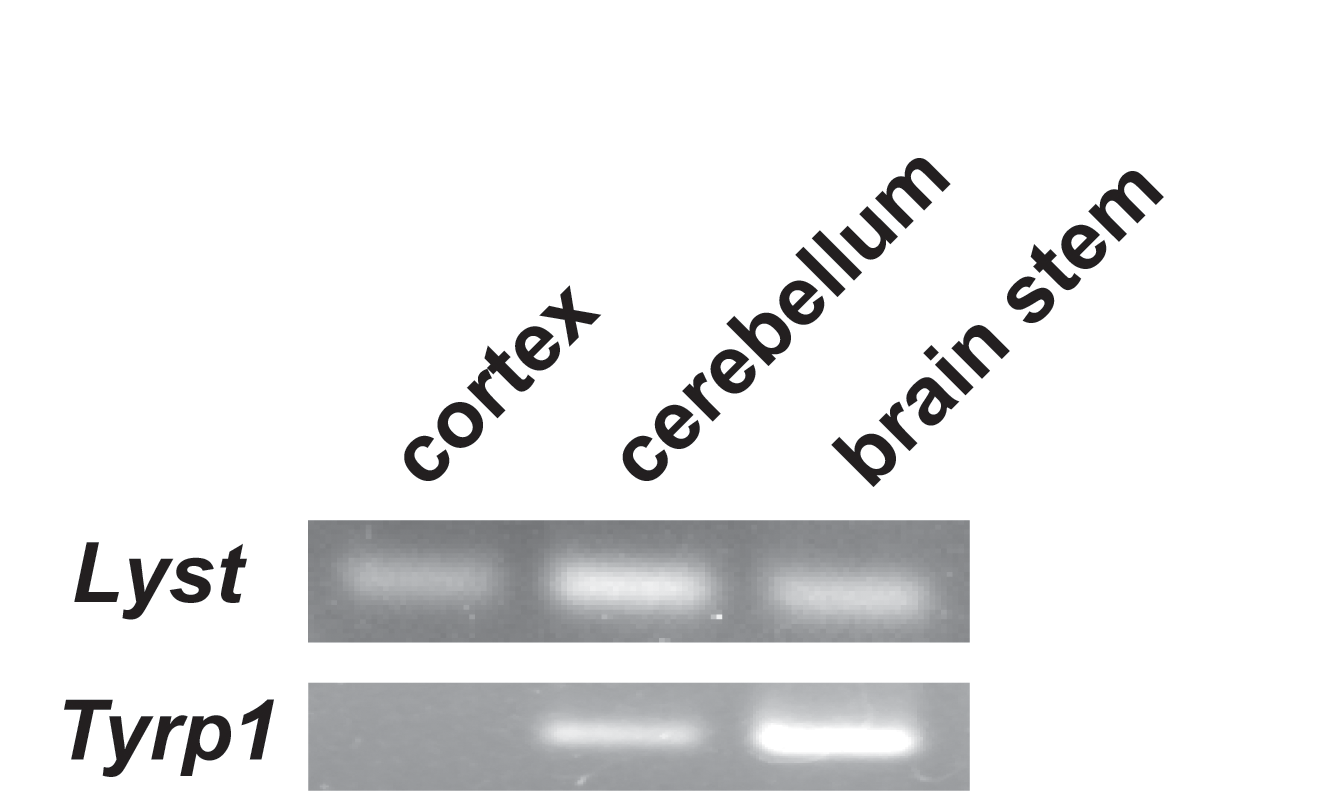

Supplement: Figure S7 — Lyst and Tyrp1 are expressed in the mouse brain. RT-PCR analysis shows Lyst expression in the cerebral cortex, cerebellum, and brain stem (top panel), and Tyrp1 expression in the cerebellum and brain stem but not the cerebral cortex (bottom panel). (0.20 MB TIF) [file pgen.1001008.s007.tif]
